# LncRNA SNHG1 Promotes the Progression of Pancreatic Cancer by Regulating FGFR1 Expression *via* Competitively Binding to miR-497

**DOI:** 10.3389/fonc.2022.813850

**Published:** 2022-01-24

**Authors:** Shihong Chen, Wenyi Guo, Mingyang Meng, Dong Wu, Tao Zhou, Lei Wang, Jianwei Xu

**Affiliations:** ^1^ Department of Pancreatic Surgery, General Surgery, Qilu Hospital, Cheeloo College of Medicine, Shandong University, Shandong, China; ^2^ Department of General Medicine, Xiangyang NO.1 People’s Hospital, Hubei University of Medicine, Xiangyang, China; ^3^ Department of Gastroenterology, Qilu Hospital, Cheeloo College of Medicine, Shandong University, Shandong, China

**Keywords:** LncRNA SNHG1, MiR-497, FGFR1, CeRNA, pancreatic cancer

## Abstract

**Background:**

Long noncoding RNA small nucleolar RNA host gene 1 (SNHG1) is dysregulated in a variety of tumors. However, little is known of its role in pancreatic cancer (PC).

**Methods:**

The role of SNHG1 on PC cell proliferation, migration, invasion, apoptosis, and the epithelial-mesenchymal transition (EMT) were assessed *in vitro* using MTT, EDU, wound healing, and Transwell assays, as well as flow cytometry and western blotting. Luciferase reporter assay, western blotting, and qRT-PCR were used to examine SNHG1 regulation. Tumor growth in mice was also investigated.

**Results:**

Downregulation of SNHG1 blocked cell proliferation, migration and invasion, and induced apoptosis *in vitro*, while also inhibiting the EMT, shown by changes in the biomarkers E-cadherin, N-cadherin, and Vimentin. The opposite results were observed on upregulation of SNHG1. *In vivo* experiments showed that downregulation of SNHG1 inhibited tumor development in nude mice. Furthermore, experiments investigating the regulatory mechanism of SNHG1 indicated that SNHG1 acted as a competitive endogenous RNA, positively regulating the expression of fibroblast growth factor receptor 1 (FGFR1) through sponging miR-497. Rescue experiments demonstrated that the effects of SNHG1 downregulation on PC cells were attenuated when simultaneously inhibiting the levels of miR-497.

**Conclusions:**

SNHG1 upregulates FGFR1 expression by sponging miR-497, which promotes the progression of PC. SNHG1 may thus be a novel target for treating PC.

## 1 Introduction

Pancreatic cancer (PC) is a digestive tract tumor with a high mortality rate (1). It has been reported that PC is the third leading cause of cancer-associated death in the European Union (2). The prognosis of PC patients is notorious because the median survival time is only about six months (3). Surgery is the main treatment method. However, even after surgery, the five-year survival rate is only 10-20%. The poor prognosis of PC patients relates to the early invasion of cancer cells to adjacent tissues and metastasis (4, 5). Therefore, solving this problem may be an effective means of treating PC.

The epithelial-to-mesenchymal transition (EMT) is the process in which epithelial cells develop mesenchymal qualities (6). The downregulation of E-cadherin (E-CAD) and upregulation of other proteins such as N-cadherin (N-CAD), Vimentin (VIM), and OB-cadherin (OB-CAD) constitute significant hallmarks of the EMT (7). The EMT is considered a critical event in the metastasis of malignant tumors (8). Cancer cells lose their epithelial properties during this process and acquire mesenchymal characteristics that endow them with migratory and invasive abilities (9). There is mounting evidence that the EMT is a crucial step in the progression of PC and is closely associated with its resistance to chemotherapy (10). Early clinical data also showed that targeted EMT in PC is a promising treatment strategy (11). There are many reports that long noncoding RNAs (LncRNAs) can regulate the EMT (12).

LncRNAs are non-coding transcripts with an average length of more than 200 nt (13). Evidence shows that LncRNAs control gene expression at the transcriptional or post-transcriptional levels and thus have the potential for modifying tumor progression (14, 15). MicroRNAs (miRNAs) are a subset of non-coding RNAs that have lengths of about 22 nt. They bind to the 3’-UTRs of mRNAs through microRNA response elements (MREs) which can inhibit the translation of mRNA or lead to the degradation of mRNA. Some LncRNAs can bind to miRNAs and reduce the inhibitory effect of miRNA on mRNA; these are termed competitive endogenous RNAs (ceRNA) (16). These mechanisms are present in PC. For example, SNHG16 can regulate the miR-218/HMGB1 axis through the ceRNA mechanism to promote PC progression (17), and SNHG12 sponges miR-320b to accelerate the EMT process in PC (18).

LncRNA SNHG1 has been shown to be involved in various tumors such as glioma and prostate, breast, and bladder cancer (19–22). It has been reported that elevated SNHG1 levels are associated with poor outcomes in multiple cancers (23). More importantly, it has been found that SNHG1 is raised in PC tissues and cell lines (24). On this basis, we have investigated the functional role and potential mechanism of SNHG1 in PC.

## 2 Materials and Methods

### 2.1 Ethics Statement

The animal study was reviewed and approved by the Key Research and Development Project of the Shandong Province of China (NO.2019JZZY011008).

### 2.2 Cell Culture and Transfection

All cell lines were obtained from the Procell Life Science&Technology and authenticated by STR profiling, then cultured in DMEM (Thermo, Shanghai, China) at 37°C with 5% CO_2_. Interference of LncRNA SNHG1 was performed using siRNA (si-SNHG1-1, si-SNHG1-2) sequences synthesized by Tsingke Biotechnology (Beijing, China). For LncRNA SNHG1 overexpression, pcDNA3.1-SNHG1 and pcDNA3.1 plasmid vector (NC) were constructed by Tsingke Biotechnology. For miRNA function studies, miRNA mimics (miR497-mimics) and negative control miRNA mimics (miR497-NC) were purchased from Tsingke Biotechnology, the final concentration of the mimics was 50 nM. Cells were transfected with Lipofectamine 3000 (Invitrogen, Shanghai, China) and harvested at 72h according to the manufacturer′s instruction.

### 2.3 Quantitative Real-Time PCR (qRT-PCR)

The qRT-PCR was performed as previously described (25). Total RNA was extracted from cells using TRIzol (Vazyme, Nanjing, China). RNA quality was checked by the OD ratio (A260/A280) with a Nano-400A (Allsheng, Hangzhou, China). Subsequently, the HiScript II Q RT SuperMix kit (Vazyme) was used to transcribe total RNA as cDNA. Amplified products were detected with SYBR Green (Vazyme). GADPH and U6 were used as internal references. Experiments were conducted at least three times. The qRT-PCR data were quantified using the 2^-ΔΔCT^ method. Primers were obtained from Tsingke Biotechnology (Beijing, China), and the sequences are listed in **Supplementary Table 1**.

### 2.4 Western Blot (WB)

Total protein was extracted using RIPA lysis buffer (Servicebio, Wuhan, China) and quantified with a BCA protein assay kit (Solarbio, Beijing, China). After adding loading buffer, samples were boiled for 10 min, separated on SDS-PAGE, and transferred to PVDF (polyvinylidene fluoride) membranes. Membranes were blocked (5% skimmed milk powder in TBST for 2h at room temperature) and probed with primary antibodies overnight at 4℃. The antibodies used were against FGFR1 (Proteintech Cat# 60325-1-Ig, RRID : AB_2881435), E-CAD (Huabio, ET1702-53), N-CAD (Cell Signaling Technology Cat# 4061, RRID : AB_10694647), VIM (ABclonal Cat# A11883, RRID : AB_2772859), PARP (Cell Signaling Technology Cat# 9532, RRID : AB_659884), BAX (Cell Signaling Technology Cat# 5023, RRID : AB_10557411), BCL-2 (Cell Signaling Technology Cat# 2872, RRID : AB_10693462), and GAPDH (Proteintech Cat# 10494-1-AP, RRID : AB_2263076). Then, the membranes were incubated with IgG-horseradish peroxidase secondary antibodies (1:2000 dilution) for 2 h and finally washed three times, 10 min each time. The target proteins were detected using a Tanon 4800 imaging system (Yuanpinghao Biotechnology CO.LTD, Beijing, China) using the Western Lightning Plus ECL kit (PerkinElmer, Shanghai, China). Bands detected by Image Lab software.

### 2.5 MTT Assay and Colony Formation

Cell viability was evaluated by the MTT cell proliferation assay. About 5 × 10^3^ cells were seeded in 96-well plates. Twenty microliters of MTT solution were added after culturing for 24, 48, 72, and 96 h, after which 150 μL per well of DMSO were added to dissolve the crystals. Lastly, absorbance was measured at 490 nm in a microplate reader. Assays were conducted in triplicate (for each experiment) with three independent biological replicates.

Colony formation was used to assess cell proliferation. Cells (1× 10^3^/well) were seeded in a six-well plate. After two weeks of incubation, 1 ml of 4% paraformaldehyde was added to each well for 30 min for fixation. Then, 1 ml of crystal violet was added for staining. Finally, after washing with PBS, each well was photographed separately.

### 2.6 5-Ethynyl-2ʹ-Deoxyuridine (EDU) Assay

EDU staining was performed using the BeyoClick™ EDU Cell Proliferation Kit with Alexa Fluor 555 (Beyotime, Shanghai, China). Cells were incubated with 10 μM EDU for 2h, followed by 4% paraformaldehyde fixation for 15 min. Then, cells were washed three times in PBS with 3% BSA and permeabilized with 0.3% PBS-Triton X-100 for 15 min. Last, 500 μL of the Click Additive solution was added to each well and protected from light for 30 min. After washing three times with PBS, cells were incubated with Hoechst 33342 staining solution for 15 min. Images were acquired with a fluorescence microscope (Nikon, Tokyo, Japan).

### 2.7 Transwell Assay and Wound-Healing Assay

Cell migration was investigated using the Transwell assay. Cells (2×10^4^) in serum-free medium were seeded in the upper chamber, while the lower chamber contained medium with 10% FBS. After 24 h, the cells on the upper chamber surface were removed with a cotton-tipped swab while the cells that had migrated to the lower side were fixed and stained as above. The cells in three random fields were counted under optical microscopy.

Wound-healing experiments were used to determine the ability of cancer cells to migrate. Cells were inoculated into a six-well plate and grown until 80% confluent. The monolayer was then scraped horizontally or vertically with a 200-µl pipette tip, and the fragments were cleaned with PBS before adding culture medium. The cells were then photographed under optical microscopy, cultured for a further 24 h, and re-photographed. The same field of vision was maintained throughout the experiment.

### 2.8 Flow Cytometry

Apoptosis was examined using the Annexin V-FITC/PI apoptosis detection kit (Vazyme). FACS was performed on a BD Accuri^®^ C6 Plus (BD Biosciences, NJ, USA) and analyzed by FlowJo. Briefly, cells were collected, washed with pre-cooled PBS, and resuspended in 100μl 1× binding buffer. Five microliters of Annexin V-FIFC and PI staining solution were added to the cell suspensions and incubated for 10 min. Lastly, 400 μl of 1× binding buffer was added to the culture tube and analyzed within 1h by flow cytometry.

### 2.9 Luciferase Assay

Panc-1 cells were transfected with LncRNA SNHG1 mutant type or LncRNA SNHG1 wild-type (LncRNA SNHG1-WT) plasmid and then co-transfected with miR-497 mimics or miR-NC mimics. Similarly, the dual-luciferase vector containing wild-type FGFR1 3′UTR (FGFR1-WT) or mutant FGFR1 3′UTR (FGFR1-WUT) was established. The cells were then co-transfected with the dual-luciferase vector and miR-497 or miR-NC mimics. After incubation for 24 h, the Double luciferase reporting kit (Promega Co., USA) was used to detect luciferase activity.

### 2.10 Animal Experiments

Cells were injected into nude mice to evaluate the function of SNHG1. In brief, Panc-1 cells (3×10^6^) transfected with LV-NC-RNAi or LV-SNHG1-RNAi recombinant lentiviruses were injected subcutaneously into the flanks of the mice (n=5) and the sizes of the tumors were determined every two days, with the volume calculated as length/2 × width^2^.

### 2.11 Statistical Analysis

SPSS 21.0 software (IBM Corp., Armonk, NY, USA) was used for analysis. Figures were created in GraphPad Prism 8.0. Measurement data were expressed as means ± standard deviation (SD). Each intervention group was compared with the control group. The Student’s t-test was used for comparisons between two groups.

## 3 Results

### 3.1 The Effect of LncRNA SNHG1 on PC Cells Proliferation, Viability, Invasion, and Apoptosis

#### 3.1.1 Downregulation of SNHG1 Suppresses PC Cell Proliferation and Viability

**Figure 1:**
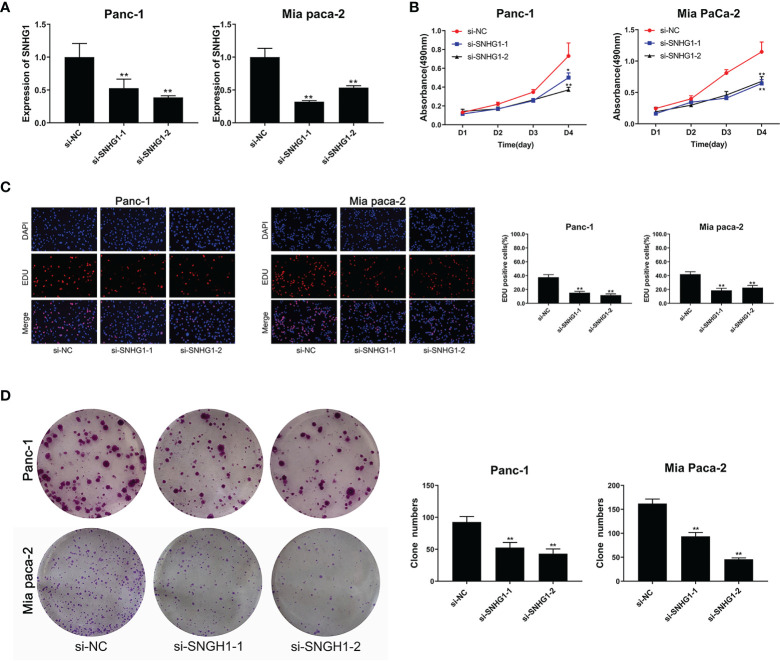
Downregulation of SNHG1 suppresses PC cell proliferation and viability. **(A)** SNHG1 expression was markedly decreased in PC cells after SNHG1 silencing. **(B)** Downregulation of SNHG1 suppressed cell viability. **(C)** EDU assay showed the reduction in PC cell proliferation after SNHG1 silencing. **(D)** Colony formation assay showed suppression of cellular proliferation with upregulated SNHG1 expression. The data represented as means ± SD of three independent experiments. *p < 0.05, **p < 0.01.

SNHG1 was silenced by transfection with si-RNA (si-SNHG1) in Panc-1 and Mia PaCa-2 cells and verified by qRT-PCR (**Figure 1A**). MTT and colony formation showed assays that silencing of SNHG1 attenuated cell proliferation (**Figures 1B, D**). EDU proliferation assays showed that cell viability was reduced (**Figure 1C**). Downregulation of SNHG1 thus reduces both PC cell proliferation and viability.

#### 3.1.2 Downregulation of SNHG1 Inhibits PC Cell Invasion, Migration and Promotes Apoptosis

**Figure 2:**
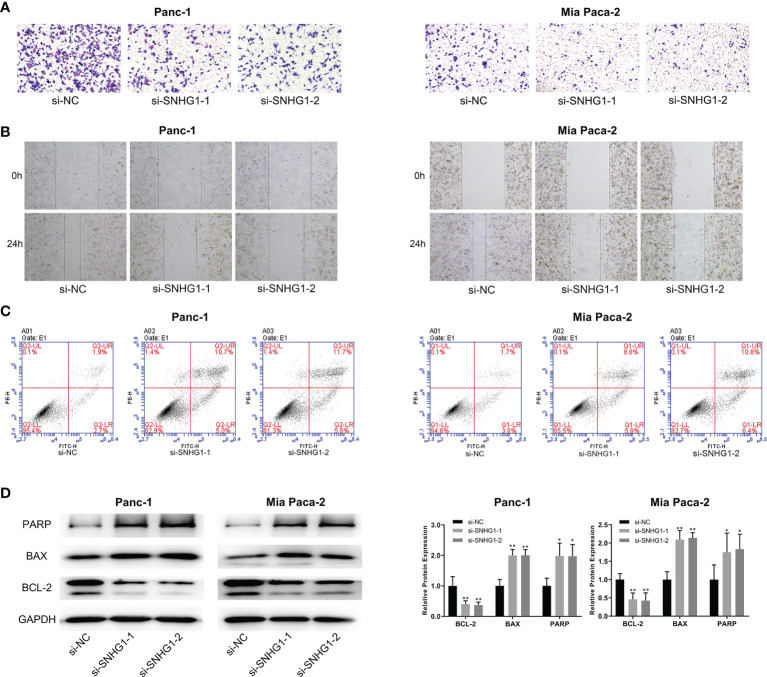
Downregulation of SNHG1 suppresses PC cell invasion and promotes apoptosis. **(A, B)** Scratch wound-healing assay and Transwell migration assay showed impaired migration and invasion ability in Panc-1 and Mia paca-2 cells after SNHG1 silencing. **(C)** Increased apoptosis after SNHG1 silencing was shown by flow cytometry. **(D)** WB of apoptosis-related proteins. *p < 0.05, **p < 0.01.

Transwell and wound-healing assays, together with flow cytometry, were performed to evaluate the role of SNHG1 in invasion, migration, and apoptosis. Downregulation of SNHG1 significantly inhibited cell invasion and migration (**Figures 2A, B**), while flow cytometry showed increased apoptosis (**Figure 2C**). The levels of the apoptosis-related proteins PARP, BAX, and BCL-2 were detected by WB (**Figure 2D**). More details are given in **Supplementary Figure 1**.

#### 3.1.3 Upregulation of SNHG1 Enhances PC Cell Proliferation and Viability

**Figure 3:**
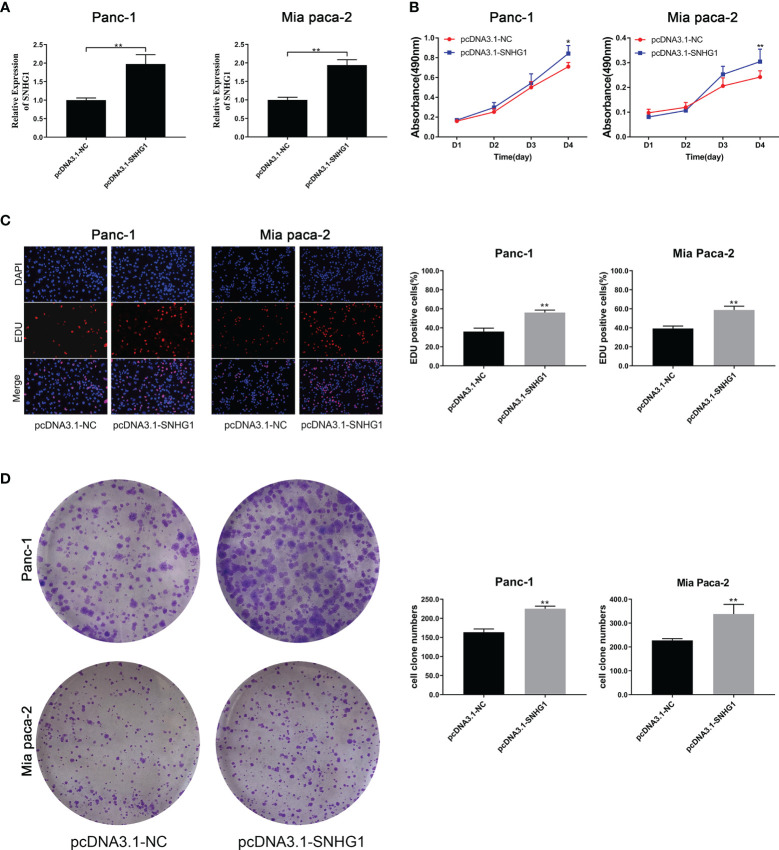
Upregulation of SNHG1 enhances PC cell proliferation and viability. **(A)** qRT-PCR quantification of SNHG1 overexpression. **(B)** Cell viability was measured by the MTT assay. **(C, D)** Cell proliferation was showed by EDU assay and colony formation assay. *p < 0.05, **p < 0.01.

To determine the effects of SNHG1 upregulation on PC cells, we conducted a reverse experiment. pcDNA3.1-SNHG1 was used to overexpress SNHG1 and qRT-PCR was used to verify the levels (**Figure 3A**). The MTT experiment showed that SNHG1 overexpression stimulated cell proliferation (**Figure 3B**), and EDU measurement showed that the PC cell viability was enhanced (**Figure 3C**). The colony formation experiments produced comparable results (**Figure 3D**).

#### 3.1.4 Upregulation of SNHG1 Promotes Invasion and Inhibits Apoptosis on PC Cells

**Figure 4:**
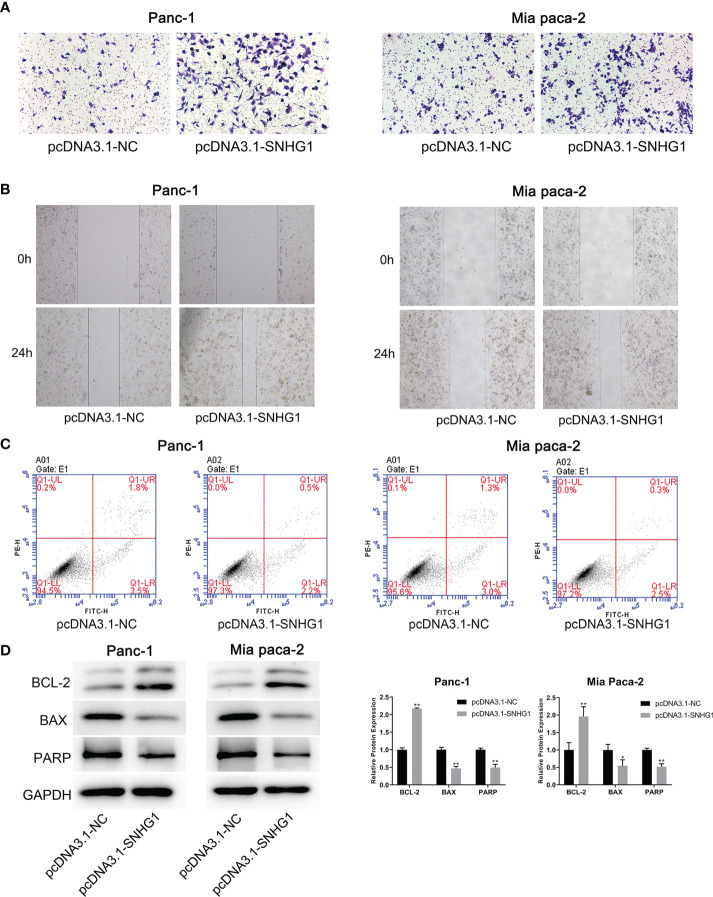
Overexpression of SNHG1 promotes PC cell invasion and migration and inhibits apoptosis. **(A, B)** Transwell and wound-healing assays demonstrated migration and invasion. **(C)** Apoptosis was determined by flow cytometry. **(D)** WB showed expression of apoptosis-related proteins. *p < 0.05, **p < 0.01.

The same methods were used to assess the migration and invasion ability of the cells. Overexpression of SNHG1 promoted cell invasion and migration (**Figures 4A, B**), whereas apoptosis was attenuated (**Figure 4C**). These results were supported by protein expression (**Figure 4D**). More details are given in **Supplementary Figure 1**.

### 3.2 LncRNA SNHG1 Promotes EMT Progression in PDAC Cells

**Figure 5:**
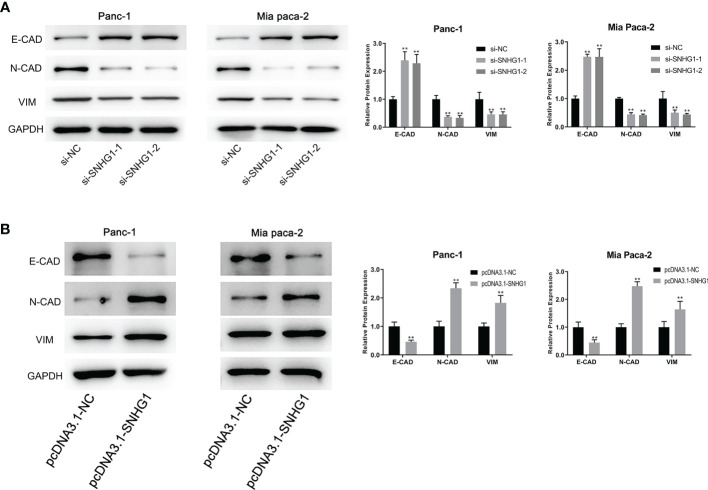
SNHG1 regulates EMT progression in PC cells. **(A)** WB showed EMT-associated proteins expression after SNHG1 silencing. **(B)** WB showed EMT-associated proteins expression after SNHG1 overexpression. **p < 0.01.

It has been reported that the EMT process is an indispensable step in tumor metastasis. E-CAD is used as the epithelial marker and N-CAD is the classical mesenchymal marker. VIM is an intermediate filament protein that plays a meaningful role in cell shape, adhesion, and motility changes during EMT. High expression of N-CAD and VIM, and low expression of E-CAD are indicative of EMT occurrence. To investigate whether SNHG1 can affect the invasiveness and migration through the EMT process, we determined the levels of EMT-related proteins. It was observed that SNHG1 silencing led to a significant elevation in E-CAD expression while the levels of N-CAD and VIM were reduced (**Figure 5A**). The opposite results were obtained when SNHG1 was upregulated (**Figure 5B**). These results suggested SNHG1 promotes PC cell migration and invasion *via* the EMT.

### 3.3 LncRNA SNHG1 Modulates FGFR1 Expression by Competitively Binding to miR-497

#### 3.3.1 LncRNA SNHG1 Sponges miR-497 and Modulates miR-497 Expression

**Figure 6:**
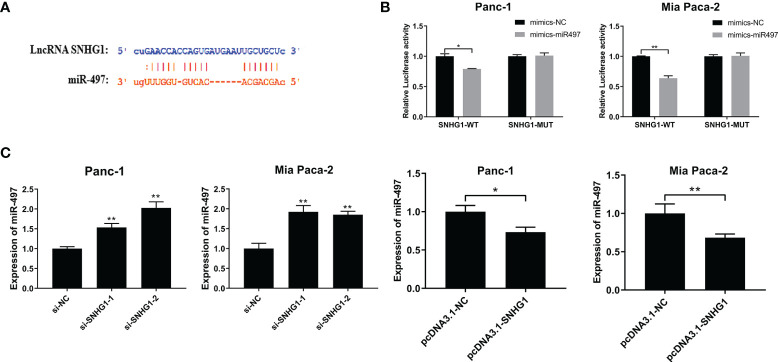
SNHG1 sponges miR-497 and regulates miR-497 expression. **(A)** TargetScan prediction of miR-497-binding sites on SNHG1. **(B)** Luciferase activity in wild-type cells after transfection with miR-497 mimics. **(C)** miR-497 expression after cell transfection with si-SNHG1 or pcDNA3.1-SNHG1 assessed by qRT-PCR. *p < 0.05, **p < 0.01.

The TargetScan database was used to predict the interaction site between SNHG1 and miR-497 (**Figure 6A**) and the Dual-luciferase reporter assay was performed to investigate the findings. The results showed that luciferase activity was significantly less in the wild-type and miR-497-mimic groups (**Figure 6B**). In addition, qRT-PCR further verified the association between SNHG1 and miR-497 after the knockdown or overexpression of SNHG1 in Panc-1 and Mia-PaCa-2 cells, demonstrating that of miR-497 levels were increased or reduced, respectively, relative to controls (**Figure 6C**). These findings indicate that lncRNA SNHG1 can bind and regulate miR-497.

#### 3.3.2 MiR-497 Targets FGFR1 and Represses FGFR1 Expression

**Figure 7:**
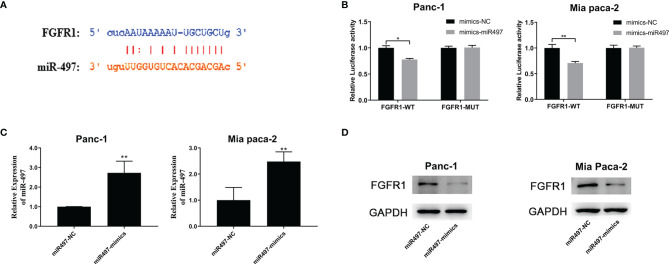
FGFR1 is targeted by miR-497 and is regulated by SNHG1 in PC cells. **(A)** TargetScan prediction of the miR-497 binding site on FGFR1. **(B)** Luciferase activity of FGFR1 wild-type was reduced after transfection with miR-497 mimics. **(C)** qRT-PCR quantification of miR-497 expression after transfection of miR-497 mimics/NC. **(D)** Reduced FGFR1 protein expression after transfection with miR-497 mimics. *p < 0.05, **p < 0.01.

The TargetScan database was also used to predict the miR-497 interaction site on FGFR1 (**Figure 7A**), and the empirical results showed that miR-497 could inhibit the luciferase activity of FGFR1-WT (**Figure 7B**), indicating FGFR1 was a target of miR-497. In addition, the miR-497 simulant was transfected into Panc-1 and Mia PaCa2 cells, and the success of the simulant construction was verified by qRT-PCR (**Figure 7C**). Interestingly, we also found that miR-497 overexpression lowered FGFR1 protein levels, shown by WB (**Figure 7D**).

#### 3.3.3 LncRNA SNHG1 Acts as a ceRNA to Regulate FGFR1 Expression

**Figure 8:**
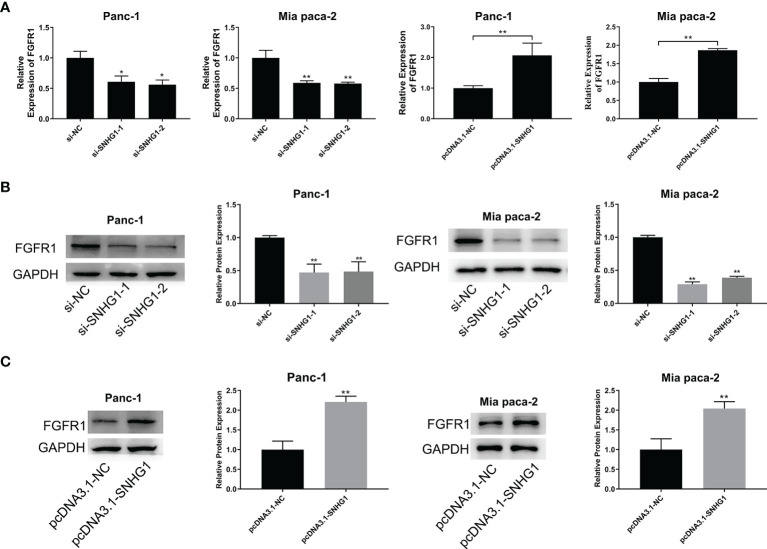
SNHG1 acts as a ceRNA to regulate FGFR1 expression. **(A)** qRT-PCR quantification of FGFR1 expression after silencing or overexpression of SNHG1. **(B, C)** WB showed FGFR1 protein levels after si-SNHG1 or pcDNA3.1-SNHG1 transfection. *p < 0.05, **p < 0.01.

To explore the link between SNHG1 and FGFR1, we next evaluated the FGFR1 mRNA level after transfection with si-SNHG1 or pcDNA-SNHG1. This showed that SNHG1 positively modulated the expression of FGFR1 at mRNA levels (**Figure 8A**). Similarly, the FGFR1 protein levels were consistent with their corresponding mRNA levels. The expression of FGFR1 followed the change of SNHG1 (**Figures 8B, C**). These results indicated that SNHG1 regulates FGFR1 expression as a ceRNA.

### 3.4 Rescue Experiment and *In Vivo* Experiment

**Figure 9:**
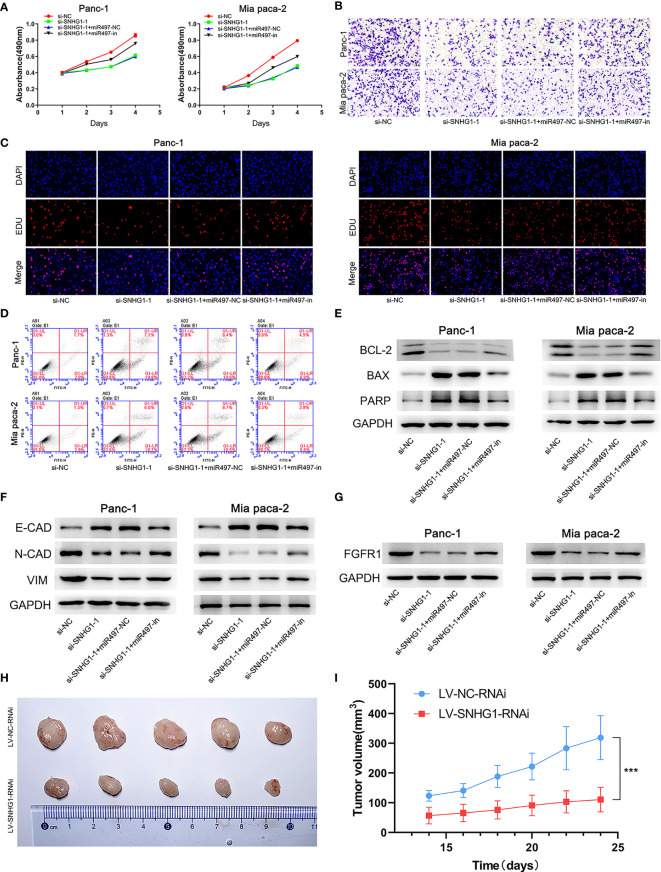
SNHG1 regulates PC cell proliferation, viability, invasion, and apoptosis *via* the miR-497/FGFR1 signaling pathway. **(A)** Cell viability was measured by MTT. **(B)** Cell invasion was measured by Transwell assay. **(C)** Proliferation was measured by the EDU assay. **(D)** Apoptosis was shown by flow cytometry. **(E, F)** WB showed expression of apoptosis-related and EMT-related proteins. **(G)** WB quantification of FGFR1 expression. **(H, I)** The influence of SNHG1 on tumor growth in nude mice. ***p < 0.001.

Four groups, si-NC, si-SNHG1-1, si-SNHG1-1+miR-497-NC (si-SNHG1+miR497-NC), and si-SNHG1-1+miR497-inhibitor (si-SNHG1-1+miR497-in), were established to explore the relationships between SNHG1, miR-497, and FGFR1. As shown in **Figures 9A–E**, the inhibition of miR-497 could mitigate the decreases in cell proliferation, invasion, and viability, as well as increased apoptosis, caused by downregulation of SNHG1 (further details are shown in **Supplementary Figure 2**). Interestingly, the EMT process, caused by the downregulation of SNHG1, was increased in the miR-497-inhibited group (**Figure 9F**). Moreover, inhibition of miR-497 could reverse the decrease of FGFR1 protein expression resulting from SNHG1 silencing (**Figure 9G**). Lastly, SNHG1 silencing resulted in a pronounced decline in the sizes and volumes of the xenograft tumors in nude mice (**Figures 9H–I**).

## 4 Discussion

PC has a high mortality rate with more than 400 000 people dying annually worldwide (26). Although surgical resection offers the only curative treatment, it is often performed too late as PC symptoms are nonspecific or asymptomatic at an early stage. In addition, most patients usually have poor outcomes due to the high rates of relapse and metastasis after surgery (27). Therefore, new approaches for the early detection and effective treatment of PC are urgently needed.

Here, it was shown that LncRNA SNHG1 significantly influenced cell proliferation, invasion, viability, and apoptosis *in vitro*. *In vivo* data provided supporting evidence for this conclusion. These findings confirm those of previous studies (28, 29). However, we have further investigated the effects of SNHG1 on cells, particularly in terms of invasion and migration. SNHG1 overexpression and silencing experiments showed that SNHG1 regulates EMT-related protein expression and regulates the EMT process, ultimately influencing the ability of the cell to migrate and invade.

Numerous studies have observed that EMT is essential for tumor progression and metastasis. During the EMT, epithelial cells lose their anchorage and ability to adhere, converting to mobile and invasive mesenchymal cells. The process can be monitored by the absence of the epithelial cell adhesion protein E-CAD and the presence of the mesenchymal proteins N-CAD and VIM (30). In this process, the downregulation of E-CAD reduces the adhesion between cells, thus enhancing cell mobility, while the increased expression of mesenchymal proteins such as VIM and N-CAD enables the neoplastic epithelial cells to develop the characteristics of mesenchymal cells, promoting tumor migration and metastasis (31). We have found that SNHG1 can upregulate E-CAD, downregulate N-CAD and VIM proteins, and promote the EMT process, while the opposite effects are seen with SNHG1 suppression.

In the next experiment, we confirmed that miR-497 has sites capable of interacting with SNHG1 and can thus serve as a direct target of SNHG1. SNHG1 competes for the miR-497 binding site through partial complementarity as a ceRNA to regulate the EMT process, affecting cell invasion and migration. We have previously found that the expression of miR-497 in cancer tissues is lower than that in normal tissues and is associated with patient prognosis (25). The rescue experiment indicated that the EMT-associated protein levels in the miR-497 inhibition group falls between the SNHG1-silenced and the control groups. Furthermore, the observed influence of miR-497 downregulation on cell proliferation and apoptosis confirmed these findings. We have confirmed that miR-497 inhibitors can partially abolish the attenuation of PC progression induced by SNHG1 silencing. Thus, the expression of miR-497 affects the competitive effects of SNHG1.

We also observed a relationship between miR-497 and FGFR1, showing that miR-497 can target and thus regulate FGFR1 expression. Mutations in the FGFR1 pathway are frequently observed in cancer (32, 33) and it has been reported that FGFR1 is strongly expressed in PC in association with poor outcomes. Although studies have shown that FGFR1 can modulate the AKT/S OX2 and Scr/NF-κB pathways affecting PC progression, the upstream regulators of FGFR1 remain unclear (16, 34, 35).

Our study demonstrated a new FGFR1 upstream mechanism. We showed that SNHG1 regulates FGFR1 expression through competitive binding with miR-497. Significantly, miR-497 downregulation abrogated the reduction in the malignant progression of PC cells induced by SNHG1 silencing. These findings demonstrate that SNHG1 may be a novel target for the treatment of PC in the future. However, there were some limitations in the present study. First, further *in vivo* experiments such as immunohistochemistry and further exploration of the relation between the expression of SNHG1 and the survival of patients with PC *via* clinical data are quite essential to clarify the value of SNHG1 in PC. Second, after SNHG1 regulates FGFR1 through competitively binding miR-497, the signal pathway that affects the progression of PC still needs to be further explored.

## 5 Conclusion

We demonstrate that SNHG1 may promote the progression of PC by competitively binding miR-497 to regulate FGFR1 expression. Targeting SNHG1 can reduce the malignant biological behavior of PC. SNHG1 may become a potential therapeutic target for PC.

## Data Availability Statement

The original contributions presented in the study are included in the article/**Supplementary Material**. Further inquiries can be directed to the corresponding authors.

## Ethics Statement

The animal study was reviewed and approved by the Key Research and Development Project of the Shandong Province of China (NO.2019JZZY011008).

## Author Contributions

SC, JX, and LW conceived the idea of the study. TZ and MM analyzed the data. WG and DW interpreted the results. SC wrote the paper. All authors contributed to the article and approved the submitted version.

## Funding

This work was supported by Natural Science Foundation of Shandong Province, China (ZR2017MH032), National Natural Science Foundation of China (Grant No.81900731), Key Technology Research and Development Program of Shandong (2019GSF108065), Natural Science Foundation of Shandong Province, China (ZR2020MH256), Key Technology Research and Development Program of Shandong (2019GSF108254) and The Medical Health Science and Technology Project of Shandong Provincial Health Commission (2019WS386).

## Conflict of Interest

The authors declare that the research was conducted in the absence of any commercial or financial relationships that could be construed as a potential conflict of interest.

## Publisher’s Note

All claims expressed in this article are solely those of the authors and do not necessarily represent those of their affiliated organizations, or those of the publisher, the editors and the reviewers. Any product that may be evaluated in this article, or claim that may be made by its manufacturer, is not guaranteed or endorsed by the publisher.

## Supplementary Material

Supplementary Figure 1 Effects of SNHG1 downregulation or upregulation on cell migration, invasion, and apoptosis. **(A, B)** Migration and invasion after SNHG1 silencing were shown by Transwell and wound-healing assays, respectively. **(C)** Apoptosis ratios after SNHG1 silencing. **(D, E)** Transwell and wound-healing assays showed migration and invasion in Panc-1 and Mia Paca-2 cells transfected by pcDNA3.1-NC or pcDNA3.1-SNHG1. **(F)** Apoptosis ratios after SNHG1 overexpression. The data was presented as means ± standard deviation (SD) from three independent experiments. *P < 0.05, **P < 0.01.

Supplementary Figure 2 Effects of miR-497 inhibitor and si-SNHG1 co-transfection on PC cell migration, viability, apoptosis, and the EMT. **(A)** Transwell assay. **(B)** EDU assay. **(C)** Expression of apoptosis-related proteins. **(D)** Expression of EMT-associated proteins. The data was presented as mean ± standard deviation (SD) from three independent experiments. *P < 0.05, **P < 0.01.
