# Supplementary figures and images for: LncRNA SNHG1 Promotes the Progression of Pancreatic Cancer by Regulating FGFR1 Expression via Competitively Binding to miR-497
Source: Front Oncol. 2022 Jan 24;12:813850. doi: 10.3389/fonc.2022.813850 (PMC8818711; doi:10.3389/fonc.2022.813850)

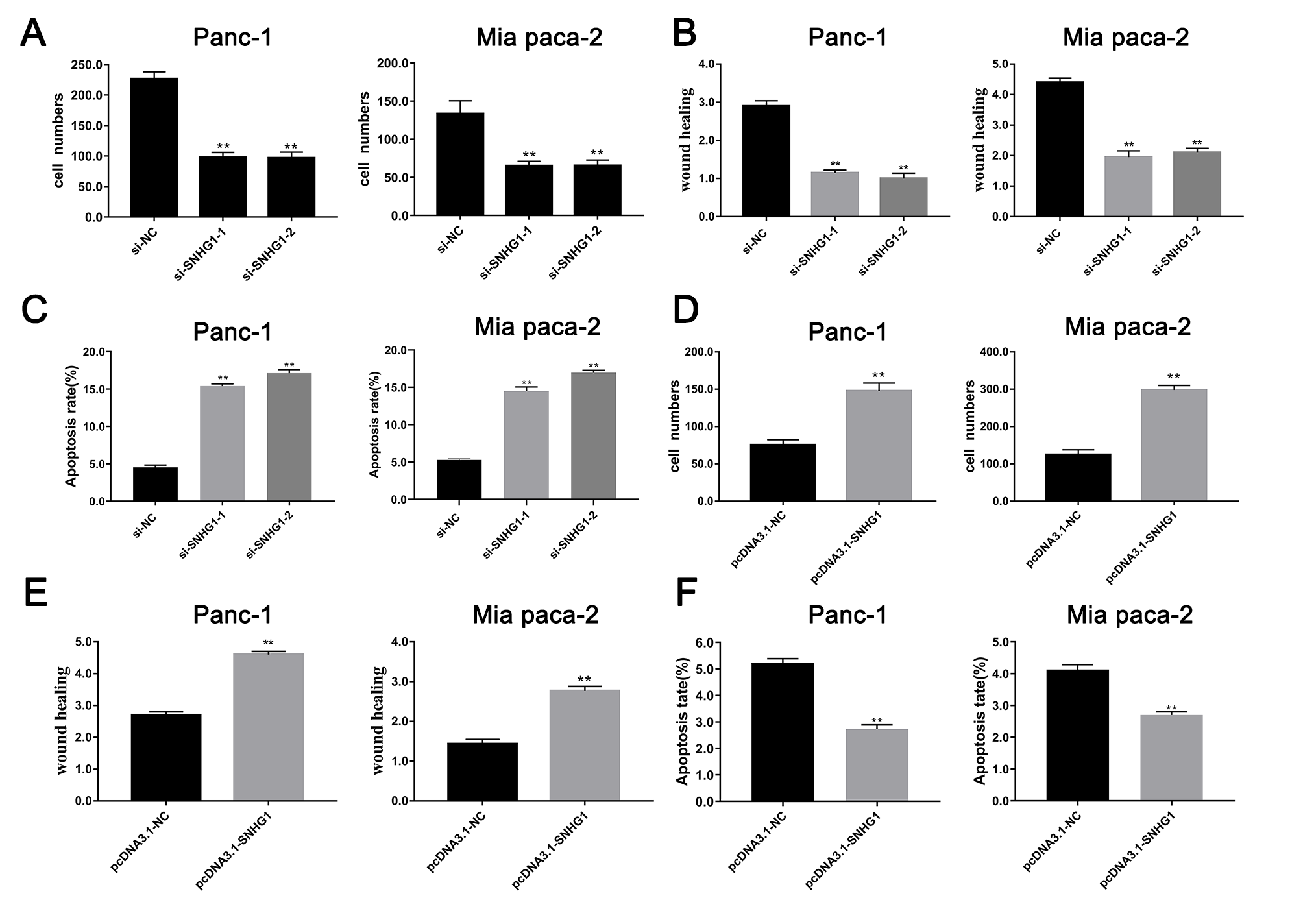

Supplement: Supplementary Figure 1 — Effects of SNHG1 downregulation or upregulation on cell migration, invasion, and apoptosis. (A, B) Migration and invasion after SNHG1 silencing were shown by Transwell and wound-healing assays, respectively. (C) Apoptosis ratios after SNHG1 silencing. (D, E) Transwell and wound-healing assays showed migration and invasion in Panc-1 and Mia Paca-2 cells transfected by pcDNA3.1-NC or pcDNA3.1-SNHG1. (F) Apoptosis ratios after SNHG1 overexpression. The data was presented as means ± standard deviation (SD) from three independent experiments. *P < 0.05, **P < 0.01. [file Image_1.tif]

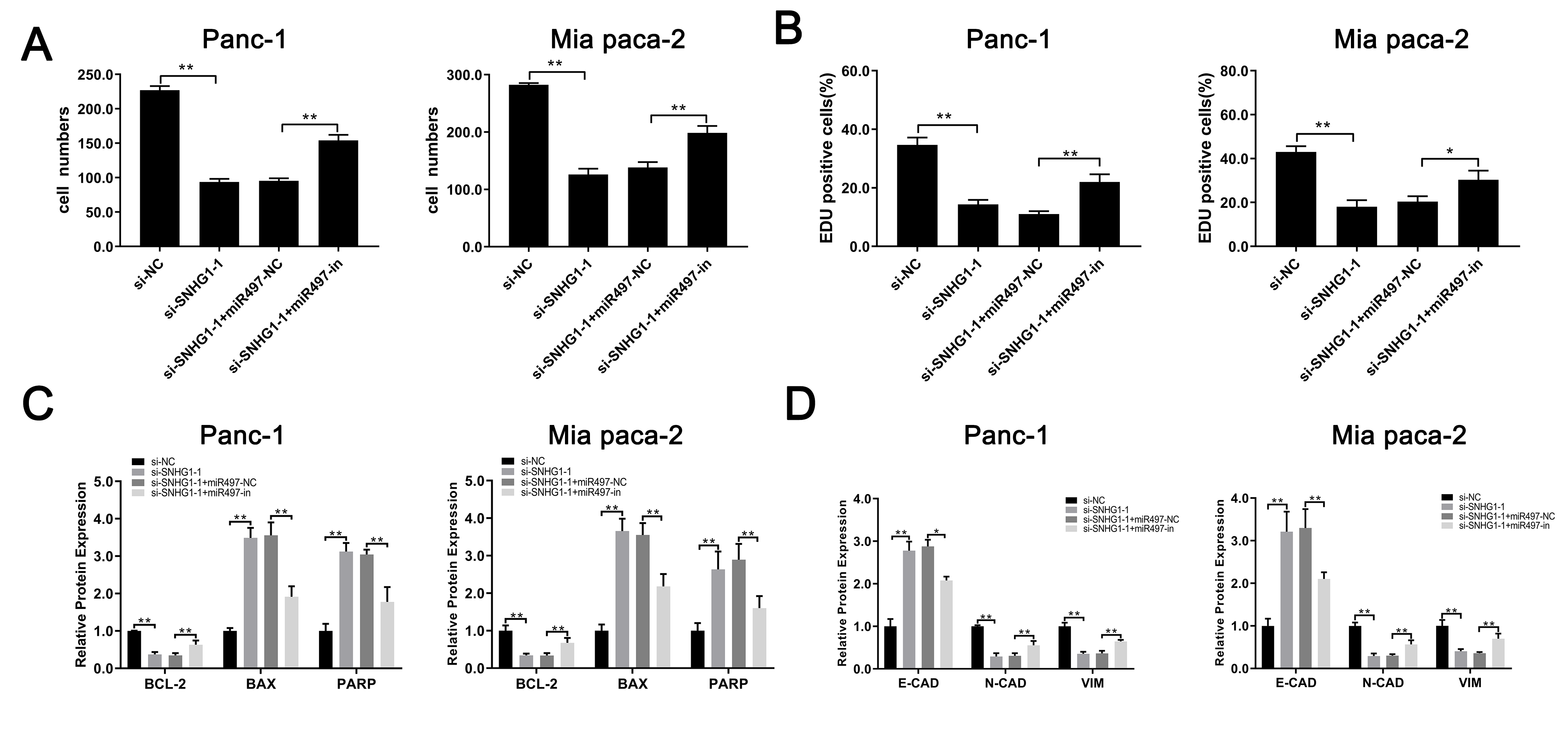

Supplement: Supplementary Figure 2 — Effects of miR-497 inhibitor and si-SNHG1 co-transfection on PC cell migration, viability, apoptosis, and the EMT. (A) Transwell assay. (B) EDU assay. (C) Expression of apoptosis-related proteins. (D) Expression of EMT-associated proteins. The data was presented as mean ± standard deviation (SD) from three independent experiments. *P < 0.05, **P < 0.01. [file Image_2.tif]
