# Supplementary material for: LncRNA SNHG1 Promotes the Progression of Pancreatic Cancer by Regulating FGFR1 Expression via Competitively Binding to miR-497
Source: Front Oncol. 2022 Jan 24;12:813850. doi: 10.3389/fonc.2022.813850 (PMC8818711; doi:10.3389/fonc.2022.813850)
Supplement: Supplementary file 3 [file Table_1.docx]

**Supplementary Table 1** Primers used in the paper were listed

| **Gene** | **Primer** | **Sequence (5′-3′)** |
| --- | --- | --- |
| **Primers for qRT-PCR** |  |  |
| lncRNA SNHG1 | forward | 5’-GCGUUACAGUAAUGUUCCATT -3’ |
|  | reverse | 5’-UGGAACAUUACUGUAACGCTT -3’ |
| miR-497 | forward | 5’-CAGCAGCACACUGUGGUUUGU -3’ |
|  | reverse | 5’-AAACCACAGUGUGCUGCUGUU -3 |
| FGFR1 | forward | 5’-AATGAGTACGGCAGCATCAAC -3’ |
|  | reverse | 5’-ACCTCGATGTGCTTTAGCCAC -3’ |
| GAPDH | forward | 5’-CGGAGTCAACGGATTTGGTCGTAT -3’ |
|  | reverse | 5’-AGCCTTCTCCATGGTGGTGAAGAC -3’ |
| U6 | forward | 5’-CGCTTCGGCAGCACATATAC -3’ |
|  | reverse | 5’-TTCACGAATTTGCGTGTCAT -3’ |
